# Effect of a mobile application-based whole-course nutrition management intervention in patients with age-related macular degeneration: a randomized controlled trial

**DOI:** 10.3389/fpubh.2026.1855870

**Published:** 2026-09-03

**Authors:** Wenmei Guo, Junli Wan, Feng Zhang, Xuemei Wu, Yan Hong, Zonghua Wang, Wei Bian

**Affiliations:** 1 Southwest Hospital/Southwest Eye Hospital, Third Military Medical University (Army Medical University), Chongqing, China; 2 Chongqing Key Laboratory of Visual Injury and Regeneration, Chongqing, China; 3 Department of Clinical Nursing, School of Nursing, Army Medical University, Chongqing, China

**Keywords:** age-related macular degeneration, dietary supplement, mobile applications, nutrition management, randomized controlled trial

## Abstract

**Aims and objectives:**

Evaluate the effect of the application (APP)-based AIM-AMD (Assessment, Introduction, Management, and App-based Maintenance Dynamically) nutrition management model in patients with age-related macular degeneration (AMD).

**Background:**

Nutritional intervention are one of the only ways to prevent the development of AMD and slow its progression. However, poor compliance with patient nutritional management leads to ineffective nutritional supplementation outcomes.

**Design:**

A randomised controlled trial was conducted from October 2023–January 2024 and performed according to the CONSORT guidelines.

**Methods:**

A total of 140 participants were randomly assigned to the intervention or control group. The intervention was administered three times per week for a period of 3 months. The intervention group was adopted with APP-based AIM-AMD nutrition management + routine nursing, while the control group was adopted with routine nursing. Comparison was conducted on the effects of different programs on AMD patient nutrient intake, nutrition knowledge, vision-related quality of life, self-efficacy and best-corrected visual acuity (BCVA) at the beginning and at the end of the intervention (Clinical Trials.gov: ChiCTR2200063978).

**Results:**

The results indicated that the intake of energy, zinc, copper, calcium, vitamin C, vitamin E and lutein was significantly higher in the intervention group than in the control group (*p* < 0.001). The intake of protein was significantly higher in the intervention group than in the control group (*p* < 0.01), whereas the intake of fat was significantly lower in the intervention group (*p* < 0.001). However, there were no significant differences in the intakes of iron, vitamin A, vitamin D, or folic acid between the intervention and control group. Vision-related quality of life, nutritional knowledge and self-efficacy scores of intervention group were higher than that of control group (*p* < 0.001). The BCVA of the intervention group did not differ significantly from that of the control group (*p* > 0.05).

**Conclusion:**

Our research demonstrates that the APP-based AIM-AMD nutrition management model offers patients comprehensive, accurate, and personalized guidance on dietary management.

**Relevance to clinical practice:**

The results of this study demonstrate that the AIM-AMD nutritional management model holds demonstrable clinical value in the nutritional management process of patients with AMD.

**Clinical trial registration:**

https://www.chictr.org.cn/showproj.html?proj=175931, Identifier ChiCTR2200063978.

## 1 Background

As identified by the World Health Organization (WHO), age-related macular degeneration (AMD) has emerged as one of the three leading blinding diseases that affect retinal degeneration and cause progressive vision impairment, accounting for 8.7% of the total cases of blindness (1). AMD is the leading cause of irreversible vision loss among older adults in developed countries (2). Given the high prevalence of blindness, AMD prevention and management have become major global public health priorities, particularly owing to the aggravation of China’s aging population. Furthermore, previous studies (3) have reported that AMD is associated with various multifactorial risk factors, such as age, genetics, smoking, and diet. Due to the complexity of the influencing factors, 65% of patients with AMD reported suboptimal improvement in visual acuity after clinical therapies, including anti-vascular endothelial growth factor treatment, photodynamic therapy, and thermal laser therapy (4, 5). Therefore, multidomain interventions should simultaneously target more factors. Several prevention studies have demonstrated the role of nutritional intervention as an important component of multidomain interventions for its effectiveness to prevent the development of AMD and slow its progression (6–9). The Royal College of Ophthalmologists (10) guidelines recommend that patients with AMD should actively supplement their nutrition or independently select over-the-counter supplements. Furthermore, the guidelines advise diets rich in fresh fruits, vegetables, eggs, and fish. Therefore, the development of comprehensive nutritional interventions to delay AMD progression is crucial.

Nutritional interventions involving dietary modifications and antioxidant supplements may delay the onset of AMD by reducing macular degeneration and increasing vision reserve. For example, the Mediterranean diet, which is popular in European countries, is characterised by a dominant amount of cereals, vegetables, fruits, and nuts, a moderate amount of fish, olive oil, and wine, and a small amount of red meat (11, 12). Greater adherence to the Mediterranean diet is associated with slower AMD progression (13). Additionally, a diet abundant in green vegetables, fish, and omega-3 rich oils along with physical exercise can slow the progression of AMD (14). A systematic review and meta-analysis by Csader et al. (15) investigated a cohort of 5,634 participants, with ages ranging from 55 to 80 years. The analysis encompassed 20 studies, of which 8 were included in the meta-analysis, comprising 414 and 216 subjects in the intervention and control groups, respectively. The results demonstrated that supplementation with lutein and zeaxanthin plus n-3 long-chain polyunsaturated fatty acids (n-3 LC-PUFA) was associated with a significant improvement in best-corrected visual acuity (BCVA) compared to the control group. Furthermore, supplementation with lutein and zeaxanthin alone significantly improved overall multifocal electroretinogram (mfERG) outcomes. The findings suggest that the combination of lutein, zeaxanthin, and n-3 LC-PUFA may be beneficial in preventing AMD progression and deterioration of visual function. A diet rich in zinc, vitamin C, vitamin E, and carotenoids has been shown to be effective in preventing and delaying AMD progression. For example, Huang et al. (16) randomly divided 112 patients age 50 years or older with early AMD into four groups: 10 mg lutein intervention group, 20 mg lutein intervention group, 10 mg lutein and 10 mg zeaxanthin intervention group, and placebo group, and continued the intervention for 2 years. The results showed that long-term supplementation with lutein (10 mg or 20 mg) or a combination of lutein and zeaxanthin significantly increased serum lutein concentration, macular pigment optical density (MPOD), and visual sensitivities in early AMD patients, whereas no improvements were observed in the placebo group. The combination of antioxidant vitamins and minerals can slow the progression of advanced AMD while preventing further vision loss. In the Age-Related Eye Disease Study Research Group (AREDS) (17) series of studies, AREDS phase 1 trial demonstrated that comprehensive nutritional preparations (vitamin C 500 mg, vitamin E 400 U, beta carotene 15 mg, zinc oxide 80 mg, and copper oxide 2 mg) were effective in delaying AMD progression. While these antioxidant vitamins and minerals are primary targets, overall ocular health and the prevention of retinal degeneration rely on a broader matrix of nutrients. Energy, protein, and fat intake reflect the general nutritional status, and dietary lipids are essential for the absorption and bioavailability of fat-soluble carotenoids like lutein and zeaxanthin (18). Essential minerals and vitamins, such as iron, calcium, and folic acid, are also crucial for maintaining retinal vascular integrity, cellular metabolism, and optic neuroprotection (19–21). Furthermore, vitamin A is indispensable for the visual cycle, and vitamin D has been shown to exert anti-inflammatory and anti-angiogenic pathways in the retina (22, 23). Therefore, evaluating this comprehensive profile of nutrients is necessary to understand the holistic impact of dietary interventions on eye health.

However, one study (24) showed that the effect of dietary intervention in reducing the risk of AMD and improving visual function is relatively small, with one possible reason being the low adherence of patients to nutritional interventions. Mobile applications (APPs) are increasingly being considered for e-health programs because of their convenience and ease of access. Telerehabilitation is an intervention that adopts a range of information and communication technologies, allowing healthcare professionals to provide remote support for the rehabilitation care of patients after hospital discharge. For instance, previous studies have reported that mobile application-based interventions have effects comparable to conventional (clinical home-based) rehabilitation on patients’ pain, physical function, range of motion, and health-related quality of life (25).

Currently, a small number of researchers have used intervention tools to monitor and guide the implementation of patient interventions, such as using mobile phones as carriers to promote the formation of patients’ dietary health behaviours, thereby improving intervention compliance (26). An APP is used to create an electronic record of the daily diet of patients with AMD, based on which the intake of various nutrients is calculated, and a nutritional assessment report is provided (26). Most of these intervention tools do not have comprehensive nutrition management concepts such as AMD-related nutrition knowledge; health education; nutrition prescription; various risk assessments; and early warning continuous tracking, monitoring, evaluation, and feedback functions. Therefore, we aimed to develop a whole-process nutrition management intervention according to the trans-theoretical model (TTM), shortened to AIM-AMD, the acronym for the six key components of the intervention. Moreover, our research team previously developed an APP (APP website: http://47.108.169.86) to provide a management platform for disease prevention, nutritional assessment, health education, nutrition prescriptions, and risk management. Therefore, this study aimed to adopt the APP to conduct AIM-AMD interventions. This article describes how the TTM framework was used to develop an AIM-AMD intervention and examine the effectiveness of AIM-AMD in promoting the adoption and maintenance of a healthy diet in patients with AMD.

## 2 Methods

### 2.1 Trail design and ethical considerations

This was a parallel-group randomized controlled trial conducted from October 2023 to January 2024. The intervention lasted for 3 months, during which participants logged their dietary intake three times per week via the APP, and researchers provided personalized feedback and support on a weekly basis. The study protocol was approved by the hospital’s institutional review board [(A) KY202287]. The study was conducted in compliance with the Declaration of Helsinki and reported in accordance with the Consolidated Standards of Reporting Trials (CONSORT) guidelines. Written informed consent was obtained from all participants, and all data were guaranteed to be confidential. The participants had the right to withdraw from the study at any time.

### 2.2 Participants

One hundred forty Participants were recruited from the ophthalmology department at four tertiary hospital located in Chongqing, the provincial capital city in Southwest China, from October 2023 to January 2024. The eligibility criteria for the study were as follows: (1) patients aged ≥18 years and clinically diagnosed with AMD (10); (2) had the best corrected visual acuity in the better eye of at least 0.3; (3) had conscious mental status and proficiency in reading and writing in the Chinese language; (4) confirmed the current absence of anti-anxiety or antidepressant medication; (5) had a smartphone with iOS or Android system; and (6) were able to provide informed consent. The exclusion criteria were as follows: (1) history of other ocular diseases or surgeries that may impact vision; (2) diagnosis of severe cognitive impairment or psychiatric disorders; (3) coexisting serious systemic conditions, such as diabetes and gastrointestinal diseases; (4) participation in any other nutritional interventions within the preceding 3 months; and (5) inability to complete the full intended duration of the intervention.

The required sample size was estimated using the formula for comparing two means, *N* = 2[(Z*α*/2 + Z*β*)/d]^2^. We set α = 0.05 (two-tailed, Zα/2 = 1.96) and power (1–β) = 0.80 (Zβ = 0.84) (27). Based on target intake levels and expected variability, the minimum detectable difference (MDE) and standard deviation (SD) were determined for five key nutrients, and the effect size d (Cohen’s d) was calculated for each. For vitamin C, an MDE of 100 mg with SD ~ 150 mg gives d ≈ 0.667; for vitamin E, MDE 80 IU, SD ~ 120 IU, d ≈ 0.667; for lutein, MDE 2 mg, SD ~ 3 mg, d ≈ 0.667; and for copper, MDE 0.4 mg, SD ~ 0.6 mg, d ≈ 0.667. Each of these yields a moderate effect size, requiring approximately 36 participants per group (total N = 72) by the above formula. Zinc showed a smaller effect (MDE 15 mg, SD ~ 25 mg, d ≈ 0.600), corresponding to about 44 per group (*N* = 88 total). Given that zinc requires the largest sample size due to its smallest effect size, this study adopts this as the most conservative estimate. It is recommended to include at least 44 participants per group (total sample size of 88) to ensure sufficient power for detecting differences in the primary nutrient indicators. Assuming a 20% attrition rate, the estimated sample size was at least 110.

### 2.3 Randomization and blinding

The eligibility of potential participants was rigorously evaluated by skilled clinical nurses, who subsequently referred them to other independent researchers. The independent researcher then offered a comprehensive explanation of the study to the potential participants and invited them to participate. An informed consent form was signed by the participants who provided permission to participate in the trial. After completion of baseline assessments, eligible participants were randomly assigned in a 1:1 ratio to either the nutritional intervention group or the control group. The random allocation sequence was generated by a research assistant not involved in participant recruitment or outcome assessment, using a computerized random number generator (Research Randomizer website: https://www.randomizer.org/) with a block design (block size of 6) and no stratification. To maintain allocation concealment, the study products and corresponding group assignments were enclosed in sequentially numbered, opaque, sealed envelopes by another research assistant not involved in the study. The envelopes were prepared in advance and kept in a secure location. When a participant was ready for randomization, the enrolling researcher opened the next envelope in numerical sequence and informed the bedside nurse of the assigned group, who then administered the corresponding intervention. Due to the nature of the nutritional intervention, neither the participants nor the nurses delivering the intervention were blinded to treatment assignment. However, the outcome assessors who performed the follow-up evaluations and the statisticians who conducted the data analyses remained blinded to group allocation throughout the study.

### 2.4 Intervention

#### 2.4.1 Model for intervention development

The TTM is one of the most popular models for guiding the development of interventions to promote healthy lifestyles. This study adopted this model because it originated from an analysis of 18 psychotherapeutic systems that identified common processes through which individuals could change their behaviours (28). Five stages of behavioural change were identified: pre-contemplation, contemplation, preparation, action, and maintenance (29). In addition to the stages of change, self-efficacy and decisional balance are the two other main components of the model that suggest primary determinants of behavioural change. The “decisional balance” indicates that behavioural change only happens when benefits of a new habit (the pros) outweigh the drawbacks and costs of doing so. “Self-efficacy” represents an individual’s readiness and sense of assurance in performing a new behaviour.

According to previous studies, TTM-tailored interventions are more successful than conventional therapies in promoting long-lasting behavioural change (30, 31). For example, Samoggia et al. (32) created a mobile nutrition intervention based on TTM and assessed its effectiveness. The findings indicate that TTM-based nutrition information apps can effectively address consumers’ personal barriers to purchasing healthy foods, enabling them to plan actions conducive to healthy eating by fostering motivation. Furthermore, the use of nutritional information APPs enhances individuals’ perceptions of healthy eating, resulting in increased self-perceived energy and confidence when encountering healthy food options. Therefore, this study aimed to determine the efficiency of TTM-based health education interventions in encouraging lifestyle changes in individuals with AMD.

#### 2.4.2 The AIM-AMD intervention

**Figure 1:**
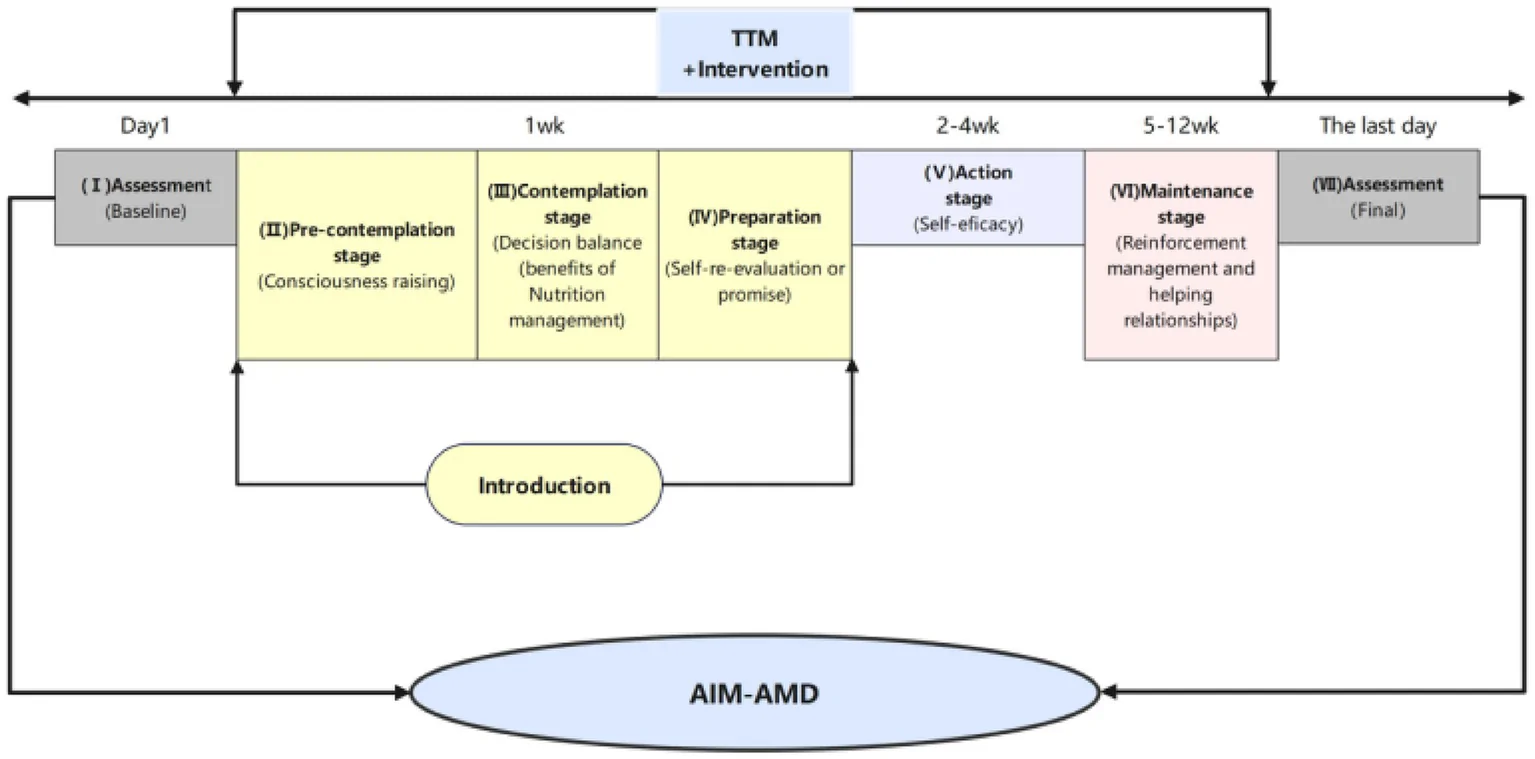
The AIM-AMD intervention based on TTM framework. (A) Assessment; (I) Introduction; (M) Management; (A) App-based; (M) Maintenance; (D) Dynamically.

The AIM-AMD intervention reflects the behavioural change process in comprehensive nutritional management. This model includes a baseline assessment, an intervention based on the TTM model, and a final evaluation. Personalised nutritional recommendations were provided based on the participants’ disease stage or the need to enhance awareness and sustain habitual changes. As shown in Figure 1. The intervention is implemented according to the TTM and the intervention elements based on the application of mobile medical intelligence, as shown in Supplementary Table S1. The intervention comprised seven sequential components (I–VII), encompassing the five behavioral change stages (pre-contemplation, contemplation, preparation, action, and maintenance) along with the baseline and final assessments, as detailed below: (I) Baseline assessment. The basic information of participants was recorded on the first day of admission, and the nutritional status of patients was assessed by clinical nursing staff. (II)Pre-contemplation stage. Participants may be ignorant of nutritional supplements or lose confidence after repeated attempts to change their nutritional supplementation behaviour. Helping participants identify the reasons for not taking nutritional supplements will stimulate their willingness to take supplements and motivate them to establish a healthy diet for AMD. This component also includes introducing patients to the relationship between AMD and nutrition, the benefits of nutritional supplementation, and the precautions for the intervention. (III) Contemplation stage. At this stage, participants are willing to adopt a diet to delay the progression of age-related macular degeneration disease, which includes supplementing with a diet rich in green vegetables, fish, and omega-3 fatty acids, as well as following a Mediterranean diet, and they plan to take action within a week. Researchers provided AMD nutrition knowledge lectures and distributed the “Dietary Nutrition Education Manual for Patients with Senile Macular Degeneration” to the patients. The participants were informed about the purpose of nutrition management and related nutrition knowledge. The importance of nutritional supplement for AMD was emphasised to help the participants establish the concept of reasonable diet and enhance their awareness of nutrition management. To address potential reluctance or low motivation to progress, researchers utilized motivational interviewing (MI) techniques. If a participant expressed hesitation, skepticism, or low readiness to change their diet within the week, the researcher did not force progression. Instead, they spent additional time exploring the participant’s specific barriers (e.g., taste preferences, cost, or family eating habits) and reinforcing their intrinsic motivation. (IV) Preparation stage: At this stage, participants were ready to immediately adopt the dietary plan of dietary intervention. Problems in the process of establishing dietary supplements as well as the plan to revise dietary supplements were discussed with the participants. Participants and their families were taught how to install and use the nutrition intervention APP. Moreover, they were also taught to independently complete the dietary nutrition recording cycle once (two working days and one weekend); The nutrition intervention goals were developed in collaboration with the participants, aiming to increase the five core nutrients (lutein, vitamin C, vitamin E, zinc, and copper) compared to baseline levels at the start of the intervention. Regarding total energy and the three major energy-supplying nutrients (carbohydrates, proteins, and fats), the targets were to increase total energy and protein intakes, decrease fat intake, and maintain carbohydrate intake at the baseline level. Participants were subsequently assisted in configuring their personalized nutritional targets and prescriptions within the dedicated application. For participants who exhibited reluctance, struggled with the APP interface, or felt overwhelmed by the nutritional targets, researchers provided one-on-one technical troubleshooting and adjusted the dietary goals to be more gradual (e.g., focusing on one nutrient at a time) to build self-efficacy and ensure they felt prepared before proceeding. (V) Action stage: At this stage, dietary management behaviour began, and participants completed diet records three times per week (twice on weekdays and once on a weekend day) in the APP until the end of the intervention. Based on the evaluation results of the dietary records, the researchers sent personalised nutrition recommendations. Health education information was sent to the participants once a week. Feedback on participants’ dietary information, such as factors affecting dietary compliance, reasons for unrecorded dietary intake, and the status of dietary knowledge acquisition, is collected weekly. Supervision reminders were sent to the participants and they were asked about their dietary change behaviours to help them find out personalised problems and improve their enthusiasm for rational diet. To manage non-progression or poor compliance during this stage, researchers initiated telephone follow-ups for participants who failed to log their diet or meet their targets. During these calls, researchers identified specific barriers (e.g., forgetfulness, lack of family support, or technical issues) and collaboratively developed practical solutions (e.g., setting phone reminders or involving family members in meal preparation) to help them sustain the behaviour change. (VI) Maintenance stage: At this stage, the maintenance stage constituted the final, consolidative phase of our behavioral intervention, grounded in principles from the Transtheoretical Model and Relapse Prevention Theory. Its objective was to stabilize the newly adopted AMD diet and mitigate the risk of regression to previous unhealthy patterns. Participants who demonstrated initial adherence by the end of the action stage transitioned to this maintenance phase, where they were encouraged to sustain the AMD diet for a minimum of 1 month, a period selected to support the transition from deliberate action to automated habit formation. To achieve this, we deployed a dual-strategy intervention: (1) Reinforcing Social-Cognitive Supports: We facilitated ongoing structured interactions between patients and healthcare providers, as well as among patient peers. Specifically, adherence was monitored weekly; participants who struggled to meet the dietary goals received additional personalized telephone counseling and barrier-resolution support from the research team, whereas those who met the goals received standard weekly reminders. These sessions served to reinforce self-efficacy, address barriers, and provide normative feedback, thereby strengthening commitment. (2) Optimizing the Behavioral Environment: We engaged participants’ families to modify the home food environment by displaying theory-informed visual prompts (e.g., slogans highlighting benefits or steps of the AMD diet) in conspicuous locations like kitchens. This tactic leveraged environmental cueing to make healthy choices more salient and automatic.

Crucially, the intervention was personalized and adaptive. The application of these strategies was continuously refined based on individual compliance data (from weekly check-ins and logs) and qualitative feedback regarding perceived difficulties or needs. This feedback loop allowed for real-time customization of support, enhancing its relevance and potential efficacy for long-term maintenance. (VII) Final assessment. On the last day, nutritional assessment and intervention effect evaluations were repeated.

### 2.5 Control group

The control group received the usual care. The researchers first sent a letter of gratitude to the participants expressing gratitude for their involvement in the study. They then provided educational materials on AMD disease nutrition management through nutrition supplement manuals, retinal disease lectures, and nutrition education seminars. During the 12-week (3-month) intervention period, the researchers conducted three monthly individual phone consultations to answer general dietary questions and remind participants to undergo regular outpatient checkups and follow-up visits. The call duration was kept to a minimum to reduce any discomfort caused by prolonged discussions on AMD issues. Usual care was provided by the same nurses as those in the intervention group.

### 2.6 Measures

#### 2.6.1 General information

A questionnaire developed by the research team was used to collect patient sociodemographic information and clinical specialty indicators. Sociodemographic data, including age, sex, marital status, and education level, were collected via patient interviews, whereas clinical specialty indicators, including duration of illness, number of eyes affected, disease category, and disease stage, were extracted from patients’ electronic medical records.

#### 2.6.2 24-h dietary recall table

The 24-h dietary recall method, abbreviated as the 24-h recall, was first used by American nutritionist Wiehl in the early 1940s (33). It was later adapted and validated by Li (34). This method is straightforward, easy to implement, and widely used for the rapid assessment of individual dietary intake. Participants are required to recall and describe all foods and beverages consumed within a 24-h period, including their types, quantities, specific times, and locations of consumption. In this study, uniformly trained interviewers conducted either face-to-face or telephone interviews to guide and assist participants in recalling and detailing all foods and beverages consumed during the past 24 h (from midnight to midnight). This included the types of items, detailed quantities (estimated with the aid of food models and utensil atlases), as well as the timing and location of consumption. To more accurately estimate individual usual nutrient intake levels, multiple (a total of three) 24-h recall surveys were conducted on non-consecutive days during the intervention period for each participant (including two weekdays and one weekend day). The investigation primarily focused on the names, quantities, ingredients, and cooking methods of foods consumed during breakfast, lunch, dinner, and snack times. Based on this information, the participants’ average daily nutrient intake was estimated. Specifically, the nutritional variables derived from the dietary recall included the daily intakes of energy, carbohydrate, protein, fat, copper, zinc, iron, calcium, lutein, folate, and vitamins A, C, D, and E. To calculate these nutrient intakes, the recorded dietary data were input into the age-related macular degeneration (AMD) dietary nutrition management module of the CED Health Management System, a software platform developed by our research team. This system automatically converted food consumption into specific nutrient intakes based on the China Food Composition Table (6th Edition) (35). Currently, in China, this method is mainly used to assess the nutrient intake of different populations (e.g., children, older individuals, and pregnant women) (36) and for large-scale health surveys to analyse the relationship between diet and health (37).

#### 2.6.3 Questionnaire on nutritional knowledge of patients with AMD

The scale was independently developed by our research team and was designed to assess dietary choices specifically for patients with AMD (38). It focuses on the selection of essential nutrients and antioxidants required for managing AMD as well as beneficial nutritional behaviours. The scale includes both single- and multiple-choice questions for a total of 16 items. A correct answer earns 1 point, whereas incorrect selections are scored 0 points, with the total score ranging from 0 to 16. A higher score indicated better knowledge of disease-related nutrition among patients with AMD. Cronbach’s alpha was 0.776. The scale has been successfully applied in a nutritional status survey (39) and in the construction of a nutritional decision aid program (40), demonstrating its applicability.

#### 2.6.4 National eye institute visual function questionnaire

The scale was developed by Mangione et al. (41), and was later adapted into Chinese by Wang et al. (42). The scale comprises 12 dimensions and 25 items, with Cronbach’s alpha coefficients for each dimension ranging from 0.73 to 0.87. The items were scored using a 5-point Likert scale, with scores ranging from 0 (worst visual function) to 100 (best visual function). Higher scores indicate better visual function. In this study, this scale was used to assess the participants’ vision-related quality of life.

#### 2.6.5 General self-efficacy scale

The scale was developed by Schwarzer and Jerusalem (43), initially consisting of 20 items, which were later reduced to 10 items and is a unidimensional scale. It was adapted and validated by Wang et al. (44). Cronbach’s alpha was 0.776. Each item is scored on a four-point scale: 1 = completely incorrect, 2 = almost correct, 3 = generally correct, and 4 = completely correct. The total score ranges from 10 to 40, with lower scores indicating lower self-efficacy.

#### 2.6.6 Best-corrected visual acuity (BCVA)

Best-corrected visual acuity (BCVA) was measured monocularly for each eye (starting with the right eye) using a standard logarithmic visual acuity chart (E-chart) at a testing distance of 5 meters, in accordance with the Chinese National Standard (GB 11533-2011) (45). Participants wore their habitual distance corrective lenses during testing. The chart features a logarithmic progression, with a ratio of $\sqrt[{10}]{10}$ between adjacent lines (equivalent to a 0.1 logMAR step) (46). Participants were required to identify the orientation of the ‘E’ optotypes (up, down, left, or right). Visual acuity was initially recorded using the 5-point scale (L), where 5.0 corresponds to 20/20 (0.0 logMAR), 4.7 to 20/40 (0.3 logMAR), and 4.0 to 20/200 (1.0 logMAR). For statistical analysis, the 5-point scale scores were converted to logMAR values using the formula (47): “logMAR” = 5.0-L. For participants unable to recognize the largest optotype at 5 meters, visual acuity was sequentially assessed and recorded as counting fingers (CF), hand motion (HM), or light perception (LP) (48). All measurements were performed by trained examiners under standardized indoor lighting conditions.

### 2.7 Data collection and analyses

Data were collected by one researcher using a questionnaire. The researcher explained the purpose, significance, and procedures of the study to the patients and obtained informed consent. Paper questionnaires were distributed, and for some patients (such as those who were illiterate), the researcher read the questions aloud and provided their responses while recording the answers. The completed questionnaires were collected onsite and reviewed for completeness. Any missing information was promptly filled in. A total of 140 questionnaires were distributed, and 137 valid questionnaires were returned, yielding an effective response rate of 97%. Baseline data were collected prior to the intervention. At the end of the 3-month intervention, data on nutrient intake, nutritional knowledge, vision-related quality of life, self-efficacy scores and BCVA were collected.

Analysis of covariance (ANCOVA) was performed using SPSS 27.0 to determine whether there were statistically significant differences between the two groups and the 18 outcome measures. These measures included: (1) dietary intake (14 variables: energy, carbohydrate, protein, fat, copper, zinc, iron, calcium, lutein, folate, and vitamins A, C, D, and E); (2) nutritional knowledge (1 variable: total score); (3) vision-related quality of life (1 variable: total score); (4) self-efficacy (1 variable: total score); and (5) BCVA (1 variable) while controlling for unpredictable exogenous variables. The ANCOVA model was specified as “post-intervention value ~ group + baseline value,” meaning the intervention effect was analyzed with group as a fixed factor and the baseline value as a covariate. The use of baseline covariates increases statistical power and yields a more precise estimate of the treatment effect. For each outcome variable, we report the significance probability (*p* value) for the between-group comparison, the effect size (Cohen’s d), and the 95% confidence interval. A Bonferroni correction was applied to the hypothesis testing results for the 18 outcome measures in this study to strictly control the overall significance level.

## 3 Results

### 3.1 Participant recruitment and baseline characteristics

**Figure 2:**
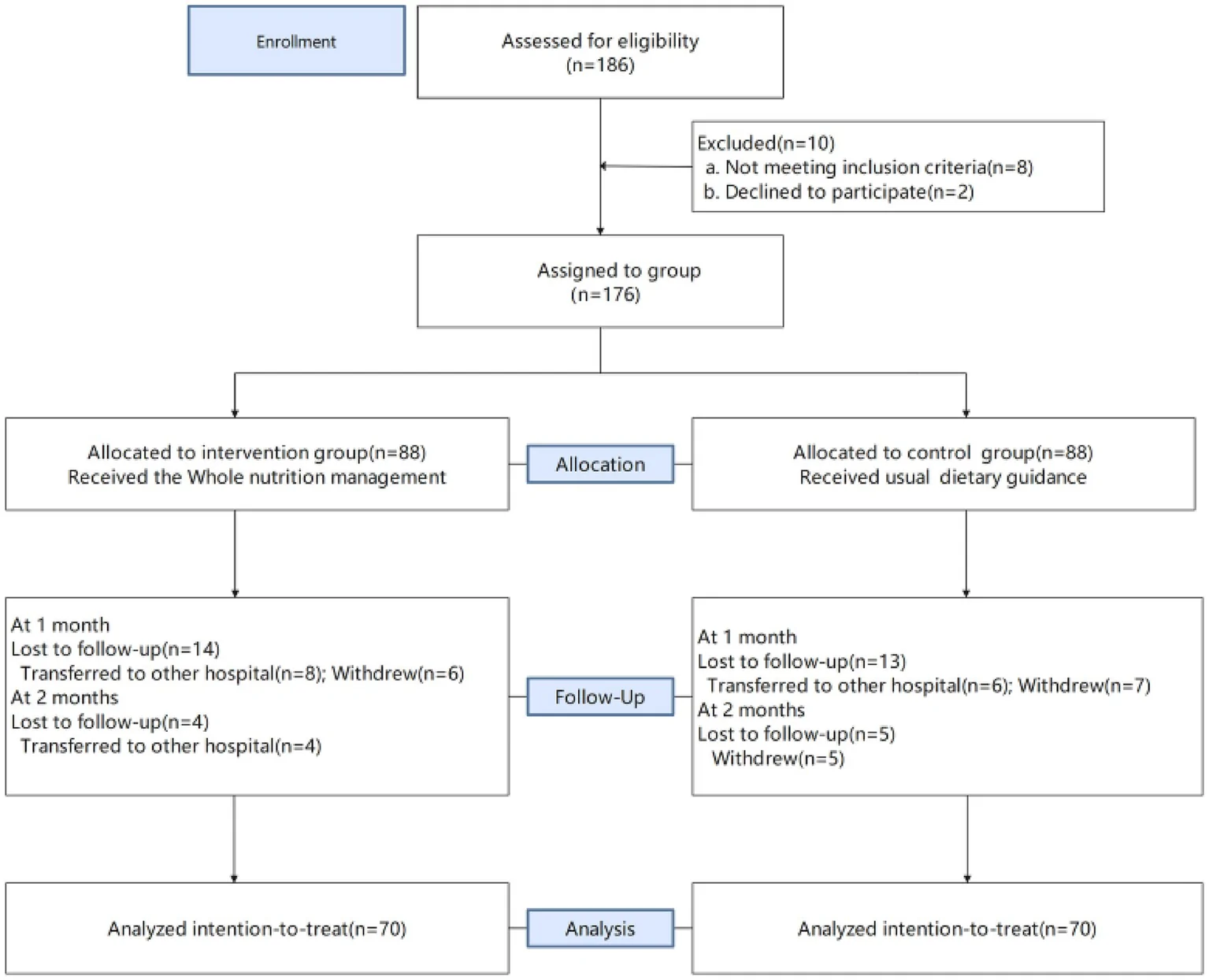
CONSORT flow diagram for participants in the study.

In total, 186 participants were recruited. However, eight participants did not meet the eligibility criteria and an additional two participants chose not to participate. Therefore, 176 eligible participants were successfully enrolled and randomly assigned to either the intervention (*n* = 88) or control (*n* = 88) group. In the intervention group, six participants withdrew after 1 month, eight participants was transferred to a different healthcare facility, and four participants were transferred to other hospitals after 2 months. In the control group, seven participants withdrew after 1 month, six participants was transferred to a different healthcare facility, and five participants withdrew after 2 months. The overall dropout rate was 20%. There were no significant differences (*p* > 0.05) in the characteristics of the participants who completed the study and those who dropped out. Ultimately, 140 participants were included in the outcome analysis with 70 participants in each group (control and intervention). A flowchart illustrating the recruitment process and the specific reasons for participant dropout is shown in Figure 2.

In the intervention group, the participants had an average age of 60.44 ± 10.05 years (range, 36–79 years). The study group comprised 28 men and 42 women. In terms of educational level, 33 participants had a junior high school education or below, and 37 had a high school education or above. Regarding marital status, 66 participants were married and four were divorced. The disease duration ranged from 3 to 36 months. Considering affected eyes, there were 21 and 49 cases with binocular and monocular involvement, respectively. Disease stage was classified as early (19 cases) or middle-to-late (51 cases). In terms of disease category, 61 participants had wet AMD, and nine had dry AMD. In the control group, the participants had an average age of 64.64 ± 11.14 years (range, 38–80 years). The study group comprised 29 men and 41 women. In terms of educational level, 56 participants had a junior high school education or below, and 14 had a high school education or above. Regarding marital status, 65 participants were married, and five were divorced. The disease duration ranged from 3 to 36 months. Considering affected eyes, there were 21 and 49 cases with binocular and monocular involvement, respectively. Disease stage was classified as early (eight cases) or middle-to-late (62 cases). In terms of disease category, 63 participants had wet AMD and seven had dry AMD, as shown in Table 1.

**Table 1:** Study participant characteristics at baseline.

Demographics and clinical features	Intervention group (*n* = 70)	Control group (*n* = 70)
Age(mean)	60.4	64.6
Sex *n* (%)		
Male	28 (40.0)	29 (41.4)
Female	42 (60.0)	41 (51.6)
Educational level *n* (%)		
High school and above	37 (52.9)	14 (20.0)
Junior high school and below	33 (47.2)	56 (80.0)
Marital status *n* (%)		
Be married	66 (94.3)	65 (92.9)
Divorced or single	4 (5.7)	5 (7.1)
Duration of illness *n* (%)		
Less than 1 year	24 (32.9)	20 (28.6)
1 year or more	46 (67.1)	50 (71.4)
Affected quantity *n* (%)		
binocular	21 (30.0)	21 (30.0)
monocular	49 (70.0)	49 (70.0)
Stage of disease *n* (%)		
Early stage	19 (27.1)	8 (11.4)
The middle and advanced stage	51 (72.9)	62 (88.6)
Category of disease *n* (%)		
Wet	61 (87.1)	63 (90.0)
Dry	9 (12.9)	7 (10.0)

### 3.2 Effect of the AIM-AMD intervention on dietary intake

The results indicated that among the three energy-supplying substances, the intake of energy in the experimental group was significantly higher than that in the control group (*p* < 0.001), the intake of protein in the experimental group was significantly higher than that in the control group (*p* < 0.01), whereas the intake of fat was significantly lower in the experimental group (*p* < 0.001). The intake of zinc, copper, calcium, vitamin C, vitamin E and lutein were significantly higher in the experimental group than in the control group (*p* < 0.001). However, there were no significant differences in the intakes of iron, vitamin A, vitamin D, or folic acid between the experimental and control groups, as shown in Table 2.

**Table 2:** Differences in dietary intake between groups (*N* = 140).

Variable (unit)	Intervention mean (*n* = 70)	Control mean (*n* = 70)	Difference (95% CI)	*p*-value	Cohen’s d
Energy (kcal)	1824.7	1465.9	335.9 (266.0, 405.8)	<0.001	1.61
Carbohydrate (g)	186.1	174.1	13.3 (−20.9, 47.4)	0.444	0.13
Protein (g)	64.9	59.5	5.63 (2.02, 9.24)	0.002	0.52
Fat (g)	37.9	46.6	−8.78 (−11.87, −5.69)	<0.001	−0.95
Zinc (mg)	29.0	21.1	7.87 (6.79, 8.94)	<0.001	2.45
Copper (mg)	5.1	3.9	1.16 (0.66, 1.65)	<0.001	0.79
Iron (mg)	15.0	14.9	0.05 (−0.96, 1.06)	0.916	0.02
Calcium (mg)	723.5	566.4	155.8 (121.1, 190.4)	<0.001	1.51
Lutein (mg)	11.7	7.2	4.47 (3.82, 5.11)	<0.001	2.33
Vitamin A (g)	288.6	289.7	−0.68 (−42.0, 40.7)	0.974	−0.01
Vitamin C (mg)	531.7	394.8	137.2 (122.1, 152.2)	<0.001	3.05
Vitamin D (g)	7.5	7.2	0.40 (−0.27, 1.08)	0.242	0.2
Vitamin E (mg)	294.9	173.6	121.5 (110.6, 132.4)	<0.001	3.75
Folic Acid (g)	119.7	124.3	−3.48 (−13.8, 6.8)	0.505	−0.11

The variables in Table 3 are listed in order of standard nutritional categories (energy and macronutrients, minerals, lutein, and vitamins) rather than their clinical importance to eye health.

### 3.3 Effects of AIM-AMD intervention on nutritional knowledge, vision-related quality of life, and self-efficacy

At the end of the intervention, the nutritional knowledge score of the experimental group was significantly higher than that of the control group (*p* < 0.001). Additionally, the vision-related quality of life score in the experimental group was significantly higher than that in the control group (*p* < 0.001). The self-efficacy level in the experimental group was also significantly higher than that in the control group (*p* < 0.001), as shown in Table 3.

**Table 3:** Differences in nutritional knowledge, vision-related quality of life, and self-efficacy between groups (*N* = 140).

Variable (unit)	Intervention mean (*n* = 70)	Control mean (*n* = 70)	Difference (95% CI)	*p*-value	Cohen’s d
Nutrition Knowledge (score)	28.9	15.1	13.21 (11.90, 14.52)	<0.001	3.38
Vision-related quality of life (score)	62.3	45.6	15.81 (13.56, 18.06)	<0.001	2.41
Self-efficacy (score)	28.6	17.9	10.72 (9.93, 11.51)	<0.001	4.55

### 3.4 Effects of AIM-AMD intervention on BCVA

At the end of the intervention, there was no significant difference in BCVA between the experimental group and the control group (*p* > 0.05) as shown in Table 4.

**Table 4:** Differences in best-corrected visual acuity between groups (*N* = 140).

Variable (unit)	Intervention mean (*n* = 70)	Control mean (*n* = 70)	Difference (95% CI)	*p*-value	Cohen’s d	Homogeneity of slopes (*p*)
BCVA (OD)	0.623	0.633	−0.010 (−0.040, 0.019)	0.477	−0.12	0.880
BCVA (OS)	0.634	0.664	−0.030 (−0.102, 0.042)	0.410	−0.14	0.571

## 4 Discussion

### 4.1 Implementing APP-based AIM-AMD nutrition management model for patients with AMD is necessary

AMD is a leading cause of blindness and can be classified into two main types: wet and dry (1). Currently, intravitreal injections of antiangiogenic drugs can slow vision loss in patients with wet AMD. However, patients often face the burdens of repeated injections, significant pain, and high medical costs. In contrast, dry AMD is irreversible and lacks an effective treatment. As the population continues to age, the prevalence of AMD and its associated economic and social burdens are expected to increase. Nutritional interventions play a crucial role in preventing and slowing AMD progression (49). Evidence suggests that all patients with AMD, regardless of disease severity, should receive dietary guidance to increase their intake of dark green vegetables, follow a low-glycaemic index diet, and consume fish at least twice per week. These dietary factors are independently and inversely associated with the risk of AMD development and progression (17, 50–54). Additionally, the AREDS showed that taking nutritional supplements containing doses of zinc, vitamin C, vitamin E, beta-carotene, and copper reduced the risk of developing advanced AMD by 25% (17, 53). A follow-up study by the same team (AREDS2) found that replacing beta-carotene with a 5:1 mixture of lutein and zeaxanthin may further reduce the risk of advanced AMD (53). Although considerable evidence has demonstrated the effectiveness and cost-effectiveness of nutritional interventions, there is still a gap between the best evidence and clinical practice. The main reasons include the lack of clear guidelines for both patients and practitioners, insufficient information provided to patients, misunderstandings about the relationship between diet and AMD, and the absence of effective and convenient support tools (55–62).

Therefore, establishing an innovative nutritional management model that is efficient, convenient, and practical for patients with AMD remains crucial. Holistic nutritional management involves a systematic, standardised, and individualised approach that dynamically adjusts nutritional interventions, dietary structure, and dosage. This model includes the formation of a professional nutrition support team, creation of management files, implementation of nutrition assessments, accurate formulation of intervention steps, and timely evaluation of intervention effectiveness. This emphasises the need for a multidisciplinary team to continuously monitor and adjust the nutritional support plan based on changes in the patient’s nutritional status. Leveraging technologies, such as the Internet and smartphone APPs, to provide patients with timely, sufficient, efficient, and personalised nutritional support is one of the most effective ways to achieve whole-course nutritional management. However, for patients with AMD, there are few reports on the design and implementation of a whole-course nutrition management model based on mobile medical APPs as the primary platform. Therefore, whole-course nutritional management models should be explored for patients with AMD who use mobile medical APPs.

### 4.2 Effectiveness of constructing AIM-AMD nutrition management model for patients with AMD based on an APP

#### 4.2.1 The APP-based AIM-AMD nutrition management model constructed in this study can effectively improve the nutrient intake of patients with AMD

The results of this study showed that the APP-based AIM-AMD nutrition management model can effectively improve the nutritional status of patients with AMD. After 3 months of intervention, the intakes of zinc, copper, calcium, lutein, and vitamins C and E in the experimental group were significantly higher than those in the control group. This demonstrates that the whole-course nutrition management model based on mobile medical technology can effectively enhance nutrient intake, particularly the five core nutrients, thereby improving the nutritional status of patients with AMD. This model employs a multidisciplinary team to develop a nutritional plan for patients, focusing on nutrition-related issues. Guiding patients to use the AMD nutrition management APP provides them with ample nutritional knowledge, enabling them to use their fragmented time for learning and reading, which, in turn, improves their understanding of nutrition. Most importantly, the daily meal recording and evaluation functions of the APP allow for comprehensive tracking, monitoring, and analysis of the patients’ dietary intake, especially the intake and compliance of the five core nutrients. The APP dynamically adjusts the nutritional management plan based on the patient’s specific adherence, thereby achieving better intervention outcomes and higher compliance, which aligns with the findings of Tang et al. (24). In their study, the mobile phone-based AMD “4S” nutrition intervention model significantly increased the intake of green vegetables, legumes, and nuts among patients. Their study also suggested that a longer intervention duration could more effectively improve patients’ nutritional knowledge and eating habits. Additionally, the intervention team promptly analysed, addressed, and resolved various issues reported by patients in real-time, greatly reducing the frequency and time required for patients to visit doctors. This approach not only enhances the efficiency of managing patients’ unpleasant symptoms and adverse reactions but also improves overall patient care. Our previous qualitative work revealed that patients with wet AMD suffer from prominent barriers of ability and access, including insufficient nutritional knowledge and inadequate professional follow-up support after discharge (63). Targeting these unmet clinical demands, the APP-powered AIM-AMD nutritional management model adopts information technology to deliver continuous, remote and convenient dietary guidance. While quantitative APP utilization indicators were not tracked within this trial, existing digital intervention research such as the StopDia study has verified that consistent active APP participation is vital to realizing clinical benefits (64). Despite the lack of usage statistics, the remarkable enhancements in dietary quality and intake of core ocular nutrients detected in the intervention group indicate that the AIM-AMD system maintained effective patient participation across the 3-month intervention period, which verifies its practicability and efficacy for AMD nutritional management. Accordingly, this APP-based model serves as a promising, innovative and all-round supplementary tool for clinical nutritional care of AMD patients.

#### 4.2.2 The APP-based AIM-AMD nutrition management model constructed in this study can effectively improve the self-efficacy and of patients

Self-efficacy refers to an individual’s confidence in their ability to successfully perform a desired behaviour or overcome obstacles to achieving that behaviour. It encompasses not only the individual’s capability to use these skills effectively and consistently but also their belief in their continued ability to do so. Additionally, self-efficacy is regarded as a key motivator and mediating factor in behaviour change (65). In this study, the implementation of the APP-based AIM-AMD nutrition management model in the intervention group significantly improved patient’s self-efficacy, with the difference being statistically significant. This can be attributed to the fact that traditional nutritional intervention models typically rely on basic nutritional knowledge from medical staff, phone follow-ups, and reminders but lack dynamic assessments and adjustments to the patient’s nutritional status, intervention content, and adherence. Consequently, these models tend to be less practical and accurate, allowing patients to receive knowledge and guidance passively without actively engaging in the process. This lack of subjective initiative often prevents patients from consistently applying intervention strategies, resulting in suboptimal compliance (66, 67). This study adopted an APP-based AIM-AMD nutrition management model to move beyond the traditional passive intervention approach, enabling patients to actively participate in the intervention process and foster positive cognition and awareness. Additionally, a one-on-one communication model was implemented through an ‘online consultation’ module in which a multidisciplinary team provided timely, personalised communication and answers based on each patient’s individual condition. This further supported the dynamic adjustment of the intervention content. Through repeated interactive interventions, patients with AMD gradually shifted their traditional beliefs and gained a deeper understanding of the benefits of nutritional management in AMD progression. They became more proactive in recording their dietary intake and adhering to nutritional standards based on their individual needs. The diet plan and goals were dynamically adjusted, which boosted the patients’ confidence and success, ultimately improving their self-efficacy.

Additionally, patients with AMD often experience a reduced vision-related quality of life due to impaired visual function (such as decreased visual acuity, central scotoma, and poor contrast sensitivity), which significantly affects their work, daily activities, and other social functions. In this study, the app-based AIM-AMD model effectively helped patients optimize their dietary structure and increase their intake of key ocular nutrients. By improving nutritional status and supporting self-management, this intervention contributed to the observed improvement in patients’ vision-related quality of life, helping them better adapt to daily activities despite their visual impairment.

### 4.3 The advantages of the APP-based AIM-AMD nutrition management model for patients with AMD

With the widespread adoption and continued advancement of modern electronic information technologies, mobile medicine has emerged as a new healthcare model that seamlessly integrates large databases, medical sensors, machine algorithms, and communication technologies. It’s dynamic, timely, convenient, and interactive features have made it widely used in both the healthcare industry and health management systems (68). Smartphones have been widely used as key platforms in disease intervention, including risk assessment, electronic health records, medical information retrieval, remote consultations and treatments, health education and promotion, and precision health intervention (69).

This study provides a comprehensive evaluation of the dietary nutritional status of patients with AMD and its influencing factors by integrating existing evidence. Drawing on advanced international experience, we developed a systematic and holistic mobile medical management model for AMD nutrition. The Delphi method and expert working group approach were employed to thoroughly review and refine both the management model and the design of the APP module content, ensuring the scientific and rational development of the APP’s functional modules. Efforts were made to minimise the issues arising from poor APP design (e.g., interface, user participation, ease of use, platform stability, and user behaviour) to enhance usability and practicality, ultimately achieving effective remote intervention across time, space, and regions. Furthermore, the model facilitated the timely and effective capture and analysis of patient data through background management systems. Dynamic adjustments to interventions were made based on real-time dietary behaviour data and compliance, ensuring that the nutritional management process adapts to the evolving needs of patients. A multidisciplinary nutrition management team, including doctors, nurses, and dietitians, was established to ensure efficient feedback and communication with patients and their families, promoting better doctor-patient communication and improving patient compliance. This new AMD whole-course nutrition management model based on mobile medical applications provides a structured and systematic approach to nutritional support. Compared to traditional, less structured follow-ups, this app-based platform offers a more integrated and accessible pathway for delivering dietary interventions, which is consistent with the documented advantages of mobile health (mHealth) technologies in chronic disease management (70). The significant improvements in nutrient intake and vision-related quality of life observed in the intervention group suggest that the AIM-AMD model is a promising tool for clinical application and future development. Additionally, with the continued growth of modern information technologies and the expansion of diverse social media platforms (e.g., WeChat public accounts and web browsers), selecting the most appropriate intervention platform based on patient-specific needs can significantly enhance compliance. The development of mobile medical products, such as wearable devices, physical sensors, and artificial intelligence powered food image recognition tools, to quickly capture nutrition-related information will further promote healthy behaviour among patients and is a key focus for future research.

### 4.4 Limitations and future directions

Several limitations of this study should be noted. First, the follow-up period was relatively short (3 months), which limits our ability to evaluate the long-term clinical efficacy of the intervention. Given that AMD is a chronic, progressive condition, nutritional interventions require sustained compliance to yield tangible clinical benefits. This short duration may explain why no statistically significant differences in BCVA were observed between the two groups (*p* > 0.05). Furthermore, because direct retinal outcomes-such as MPOD—were not measured, the clinical translation of these dietary improvements remains to be fully elucidated. Additionally, we did not perform a subgroup analysis based on whether patients had unilateral or bilateral AMD. Given our sample size (70 patients per group), further dividing the cohort into subgroups would significantly reduce the statistical power of the ANCOVA and increase the risk of Type II errors. Therefore, future longitudinal trials with extended follow-up (≥ 12 months), larger sample sizes to facilitate robust subgroup analyses, and objective retinal metrics are warranted to confirm the long-term therapeutic impact of this intervention. Second, this study was conducted in a single center in Chongqing, China. The specific regional dietary patterns (characterized by local culinary habits in Southwest China) and the cultural and healthcare context of the participants may limit the generalizability of our findings to populations with different dietary structures or healthcare systems. Future multi-center studies involving diverse geographic regions and cultural backgrounds are warranted to confirm the efficacy of this intervention. Third, potential attention bias (the Hawthorne effect) cannot be fully ruled out. Although we provided active education (manuals, lectures, and monthly phone calls) to the control group, the intervention group experienced higher engagement via the mobile app, which might have influenced subjective outcomes such as self-efficacy and vision-related quality of life; using a placebo app would provide a more balanced attention control. Fourth, although the final sample size met the requirements for the primary analysis, the 20% attrition rate may have reduced the statistical power, thereby limiting the ability to detect differences with small effect sizes. To mitigate these constraints, future longitudinal studies should incorporate larger sample sizes derived from conservative statistical power estimations that factor in potential dropouts. Moreover, iterative optimization of the APP’s user-driven features, coupled with targeted retention protocols, will be employed to maximize subject adherence and minimize attrition. Finally, app utilization and user engagement metrics (such as login frequency and active usage time) were not recorded in this study. Future research should incorporate backend data tracking to analyze how different user engagement patterns influence the efficacy of the AIM-AMD intervention.

## 5 Conclusion

Our study demonstrated that the APP-based AIM-AMD nutrition management model can provide patients with AMD with comprehensive, precise, and personalised nutritional guidance. This model promotes healthy eating behaviours and effectively improves nutrient intake, nutritional knowledge, self-efficacy, and vision-related quality of life. These improvements suggest that the model has the potential to support long-term disease management, although further long-term studies are needed to confirm its direct impact on disease prognosis, the risk of advanced AMD, and healthcare costs.

## Data Availability

The original contributions presented in the study are included in the article/Supplementary material, further inquiries can be directed to the corresponding authors.
