# Supplementary material for: Effect of a mobile application-based whole-course nutrition management intervention in patients with age-related macular degeneration: a randomized controlled trial
Source: Front Public Health. 2026 Sep 3;14:1855870. doi: 10.3389/fpubh.2026.1855870 (PMC13581780; doi:10.3389/fpubh.2026.1855870)
Supplement: Supplementary file 1 [file table_1.docx]

**Details of APP whole-course nutrition management intervention program based on TTM model**

| **TTM components** | **Session/Time** | **Purposes** | **Contents** |
| --- | --- | --- | --- |
| Baseline Assessment | Day1 |  | nutritional status assessment:①Input the basic information of the patient,②Patients fill in informed consent form,③Clinical nursing staff perform the assessment on patient's nutritional status. |
| Pre-contemplation stage | 1wk | Consciousness raising | -The researcher explained AMD-related knowledge, and precautions for not adjusting intervention dose without permission, stopping intervention without permission, et al. |
| Contemplation stage |  | Decision balance (benefits of Nutrition management) | -The researcher explained the purpose, content and relevant nutrition knowledge of nutrition management |
| Preparation stage |  | Self-re-evaluation or promise | -Patients and their families were taught how to install and use the nutrition intervention app. Moreover, they were also taught to independently complete the dietary nutrition recording cycle once (two working days and one weekend); Nutrition intervention goals were formulated with patients, and they were assisted to complete the setting of APP nutrition goals and prescriptions; |
| Action stage | 2-4wk | Self-efficacy | Personalized intervention:①Patients completed the record of three meals per day in the APP (three times per week, two working days and one weekend),② Researchers sent personalized nutrition recommendations to patients in the background according to the evaluation results, and supervision reminders in the APP (personalized reminder frequency was set according to the patient's compliance in the previous week),③Health education information was pushed once a week. Patient's feedback on the nutritional status of this week was collected once a week; Patient were asked about their behavioral changes. |
| Maintenance stage | 5-12wk | Reinforcement management and helping relationships | Maintenance and reinforcement: ① The content and impact of the preceding phase of behavior modification should be assessed, and the behavior change objectives should be dynamically adjusted, ② Peer education: Encourage patients to actively engage in patient activities, enabling those with high adherence to provide demonstration-based education and share successful experiences, ③ Family support: Family and peer support were strengthened to help patients change their behaviors, etc. ④ The specific content of the intervention was dynamically adjusted according to the compliance and individual demands of the patients. |
| Final Assessment | The last day |  | -Nutritional assessment and intervention effect evaluation were performed again. |
